# TRAIL predisposes non-small cell lung cancer to ferroptosis by regulating ASK-1/JNK1 pathway

**DOI:** 10.1007/s12672-024-00890-9

**Published:** 2024-02-21

**Authors:** Xiaofang Liu, Huiqian Deng, Mi Huang, Wei Zhou, Yilin Yang

**Affiliations:** Department III of Geriatrics, The Third Hospital of Changsha, No. 176, Labor West Road, Changsha, 410000 Hunan Province China

**Keywords:** TRAIL, Ferroptosis, ASK-1/JNK1 pathway, NSCLC

## Abstract

**Objective:**

Our current study aimed to assess the relationship between TNF-related apoptosis-inducing ligand (TRAIL) and ferroptosis in non-small cell lung cancer (NSCLC) development.

**Methods:**

The expression of TRAIL was detected by western blot, RT-qRCR and immunohistochemistry. The viability of NSCLC cells was analyzed by CCK-8 kit. The migration and invasion of NSCLC cells were detected by wound healing assay and transwell assay, respectively. Labile iron pool (LIP) was detected based on the calcein-acetoxymethyl ester method. Ferrous iron (Fe^2+^) and iron levels were assessed by detection kits. The levels of superoxide dismutase (SOD), catalase (CAT), and malondialdehyde (MDA) were measured using corresponding detection kits. Mice tumor xenograft models were used for the in vivo research.

**Results:**

The expression of TRAIL was reduced in H1299, NCL-H1395, and A549 cells compared with BEAS-2B cells. The up-regulation of TRAIL expression significantly reduced cell viability, invasion, and migration of H1299 and A549 cells. TRAIL reduced the expression of ferroptosis-related genes (FTH1, GPX4, and SLC7A11), increased the levels of LIP, iron, and Fe^2+^, and promoted lipid peroxidation, thereby predisposing NSCLC cells to ferroptosis. TRAIL up-regulated the expression of phosphate modification of ASK-1 and JNK. ASKI-1 inhibitor GS-4977 attenuated the effects of TRAIL on the viability, migration, invasion, and ferroptosis of H1299 cells. Furthermore, TRAIL further suppressed tumor growth and ferroptosis in mice tumor xenograft models.

**Conclusion:**

We indicated that overexpression of TRAIL induced ferroptosis in NSCLC cells and exerted anti-tumor effects. Mechanistically, TRAIL promoted ferroptosis by the activation of the ASK-1/JNK1 pathway. Our results may provide new therapeutic strategies for NSCLC.

**Supplementary Information:**

The online version contains supplementary material available at 10.1007/s12672-024-00890-9.

## Introduction

Non-small cell lung cancer (NSCLC) is a common and often fatal malignancy, and patients with advanced NSCLC have a poor five-year survival rate [1, 2]. The incidence of NSCLC depends on a variety of factors, including smoking, genetics, age, gender, and more [3]. The clinical treatment of NSCLC usually includes surgery, radiotherapy, chemotherapy drugs, and their combination [4]. However, the treatment effect and prognosis of NSCLC patients are still unsatisfactory. It is urgent to explore new anticancer strategies and targets to improve NSCLC treatment outcomes.

Tumor necrosis factor (TNF)-related apoptosis-inducing ligand (TRAIL) is a member of TNF ligand superfamily (TNFLSF) and is a type II transmembrane protein [5]. Previous studies have demonstrated that TRAIL plays a critical role in the regulation of tumorigenesis and development [6]. TRAIL has been demonstrated to induce cell proliferation and exerts an anti-apoptotic effect in some tumor cells [7, 8]. Anaplastic thyroid cancer-derived cells are sensitive to TRAIL-induced apoptosis, indicating that TRAIL is a promising therapeutic agent [9]. TRAIL receptor agonist MEDI3039 inhibits growth and metastases in triple-negative breast cancer, thereby extending animal survival [10]. Studies have indicated that TRAIL involves in the regulation of apoptosis in resistant NSCLC cells [5]. However, the role of TRAIL in NSCLC is not fully clear.

Ferroptosis is an iron-dependent and reactive oxygen species (ROS)-reliant new cell death that is distinct from autophagy and apoptosis [11]. Studies have shown that different physiological conditions and pathological stresses can activate ferroptosis in humans and animals [12]. Ferroptosis plays a key role in tumorigenesis and development [13], such as colon cancer [14], gastric cancer [15], and NSCLC [16, 17]. Ferroptosis-inducing agents enhance TRAIL-induced apoptosis and inhibition of tumor growth in colon cancer [14]. Studies have reported that sanguinarine induces ferroptosis of NSCLC cells by increasing the levels of ferrous iron (Fe^2+^) and ROS and reducing glutathione (GSH) content and glutathione peroxidase 4 (GPX4) expression, thereby playing an inhibition effect on the growth and metastasis of NSCLC [16]. Erastin sensitizes lung cancer cells to cisplatin by inducing ferroptosis through reduction of GSH and inactivation of GPX [17]. It may be an effective strategy for NSCLC treatment by targeting ferroptosis. However, it is still unclear on the relationship between TRAIL and ferroptosis in NSCLC.

In this research, we explored the effects of TRAIL on malignant phenotypes of NSCLC cells and ferroptosis in vivo and in vitro. The relationship between TRAIL overexpression and the ASK-1/JNK1 pathway was further analyzed in NSCLC. Our results may uncover the mechanism of TRAIL in NSCLC and provide a novel therapeutic target for NSCLC.

## Materials and methods

### Cell culture

The human NSCLC cell lines (A549, H1299, and NCL-H1395) and normal lung epithelial cell BEAS-2B (Cell Bank of Chinese Academy of Sciences, Shanghai, China) were cultured in RPMI 1640 medium (Thermo Fisher, Waltham, MA, USA) supplemented with 10% FBS and 100 U/mL penicillin/streptomycin at 37 °C in 5% CO_2_ and 95% humidity.

### Transfection

pcDNA3.1-TRAIL and pcDNA3.1-NC were obtained from RiboBio (Beijing, China). According to the instructions of manufacturer, A549 and H1299 cells were incubated with pcDNA3.1-TRAIL and pcDNA3.1-NC for 48 h using Lipofectamine 3000 reagent (Invitrogen, Carlsbad, CA, USA). Subsequently, the transfected cells were collected for the next experiments.

### Western blot assay

Protein was extracted from NSCLC cells using Protein Extraction Kit (Sigma-Aldrich, St. Louis, MO, USA). 10% SDS-PAGE was used to separate the samples, and protein samples were transferred to the PVDF membrane. Next, the membranes were blocked with 5% skim milk at 25 °C for 1 h, then incubated with the primary antibodies against TRAIL (1:500, ab2056, Abcam, Cambridge, MA, USA), ASK-1 (1:1000, ab45178, Abcam), p-ASK-1 (1:1000, ab47304, Abcam), JNK-1 (1:2500, ab199380, Abcam), and p-JNK-1 (1:1000, ab215208, Abcam) overnight at 4 °C. The membranes were incubated with secondary antibody conjugated by horseradish peroxidase (HRP) for 1 h at 25 °C. GAPDH was employed as a protein loading control. Target bands were detected by enhanced chemiluminescence western blotting detection reagents.

### Quantitative real-time PCR (qRT-PCR)

Total RNA from H1299 and A549 cells was extracted using RNA extraction kit (Sigma-Aldrich). RNA was reversed transcribed into cDNA using M-MLV Reverse Transcriptase kit (Takara, Dalian, China). The qRT-PCR was performed in a Real-Time System using a SYBR® Green PCR Kit (Takara) according to the instructions of manufacturer. The Mastercycler ep realplex detection system (Eppendorf, Hamburg, Germany) was used for RT-qPCR assay. The relative mRNA expression of gene was calculated using the 2^−ΔΔCT^ method. The primers are listed in Table 1.

**Table 1:** Primers for qRT-PCR in this study

Gene	Sequence from 5’-3’
TRAIL Forward	CAAGTCAAGTGGCAACTCCG
TRAIL Reverse	GTGAGCTGCTACTCTCTGAG
FTH1 Forward	CGCCAGAACTACCACCAG
FTH1 Reverse	TTCAAAGCCACATCATCG
GPX4 Forward	GAAGCAGGAGCCAGGGAGT
GPX4 Reverse	ACGCAGCCGTTCTTGTCG
SLC7A11 Forward	TGCTGGGCTGATTTTATCTTCG
SLC7A11 Reverse	GAAAGGGCAACCATGAAGAGG
GAPDH Forward	GAATTCATGTTTGAGACCTTCAA
GAPDH Reverse	CCGGATCCATCTCTTGCTCGAAGTCCA

###  Cell viability assay

The viability of A549 and H1299 cells was measured using Cell Counting Kit-8 (Meilunbio, Dalian, China). Cells were seeded into 96-well plates (1 × 10^4^ cells/well) and cultured for 24, 48, and 72 h, respectively. According to the manufacturer’s instruction, cells were added 10 µl CCK-8 reagent and cultured for 2 h. The OD value was measured at 450 nm using a microplate reader (Bio Tek, ELX800, Winooski, VT, USA).

### Wound healing assay

Cell migration was analyzed by wound healing assay. H1299 and A549 cells were plated into 6-well plates for adherent culture for 24 h. Pipette tips are used to scrape cells and create interstitial spaces. After 24 h, the migrating cells were observed and photomicrographs were instantly taken by the Leica microscope.

### Transwell assay

The invasion of H1299 and A549 cells was evaluated by transwell (8 μm pore, Corning, Inc.). The upper surface of the transwell chambers was pre-coated with Matrigel (BD Biosciences, Sparks, USA). RPMI 1640 medium containing 10% FBS was added to the lower chamber. Cells (5 × 10^4^ cells) were added into the upper chambers with serum-free RPMI-1640 and incubated for 24 h at 37 °C. Cells in the lower chamber were fixed with 4% paraformaldehyde and stained with 0.1% crystal violet for 15 min at 25 °C. Then, the invasion cells were counted by the light microscope.

### Measurement of SOD level, malondialdehyde (MDA), and catalase (CAT)

The levels of SOD, MDA, and CAT were assessed by ELISA assay. According to the instructions of the manufacturer, SOD, CAT, and MDA levels were measured using corresponding detection kit (Esebio, Shanghai, China).

### Labile iron pool (LIP), Fe^2+^, and iron assays

LIP was detected in A549 and H1299 cells based on the calcein-acetoxymethyl ester method. Cells were treated with calcein acetoxymethyl ester (2 µM) (Corning Inc., Corning, NY, USA) at 37 °C for 30 min and were incubated without or with 5 µM deferoxamine for 1 h at 37 °C. The final concentration of 100 µM deferoxamine mesylate is used to remove the iron in calcein. Then, the fluorescence at 485 nm excitation and 535 nm emissions was measured by the fluorescence plate reader (Thermo Fisher). The fluorescence change was used as an indirect measurement of LIP after the addition of deferoxamine. According to the manufacturer’s instructions, intracellular Fe^2+^ and iron levels were detected using the corresponding detection kits (Sigma-Aldrich).

### Animals

The overexpression of TRAIL (pcDNA3.1-TRAIL) and negative controls (pcDNA3.1-NC) were constructed through the lentivirus-based vectors by RiboBio (Beijing, China). BALB/c male nude mice (five weeks, 20–22 g) (SiPeiFu Biotechnology Co., Ltd., Beijing, China) were used in this experiment. H1299 cells (1 × 10^6^) transfected with pcDNA3.1-NC or pcDNA3.1-TRAIL were subcutaneously injected into the right dorsal flank of the nude mice to establish mice tumor xenograft models (*n* = 6). The mice were observed every day and the tumor volume was measured every 7 days. Mice were sacrificed through inhalation of isoflurane (Rayward, Shenzhen, China) for 2–3 min after 28 days, and the tumor tissues were collected and the tumor weight was measured. Tumor volume was calculated as (length × width^2^)/2. All animal experiments were approved by the committee of our hospital and conducted in accordance with the China Animal Welfare Legislation. The tumor size did not exceed the permitted maximum diameter of 20 mm and maximal volume of 1000 mm^3^.

### Immunohistochemistry (IHC) staining

Briefly, the tumor tissues were fixed with 4% paraformaldehyde, next wrapped with wax and cut into 4-µm slices. Then the paraffin sections were deparaffinized, rehydrated, treated with 1% citric acid buffer, blocked with 3% H_2_O_2_ and incubated with TRAIL antibody (Cat#3219, 1:800, Cell Signaling Technology, Danvers, MA,USA) overnight at 4 °C followed by secondary antibody incubation. Finally, the slides were dyed with diaminobenzidine solution and counterstained by hematoxylin, then sealed with neutral resin, and viewed through a microscope (IX71, Olympus, Tokyo, Japan).

### Statistical analysis

Statistical data were presented as the mean ± standard deviation (SD) and were analyzed with Prism 7.0 software (GraphPad, La Jolla, CA). Two-group comparisons were analyzed by Student’s t-test. Multiple group comparisons were analyzed by one-way ANOVA followed by Tukey’s post hoc test. *P* < 0.05 was considered significant.

## Results

### TRAIL is down-regulated in NSCLC cells

**Fig. 1:**
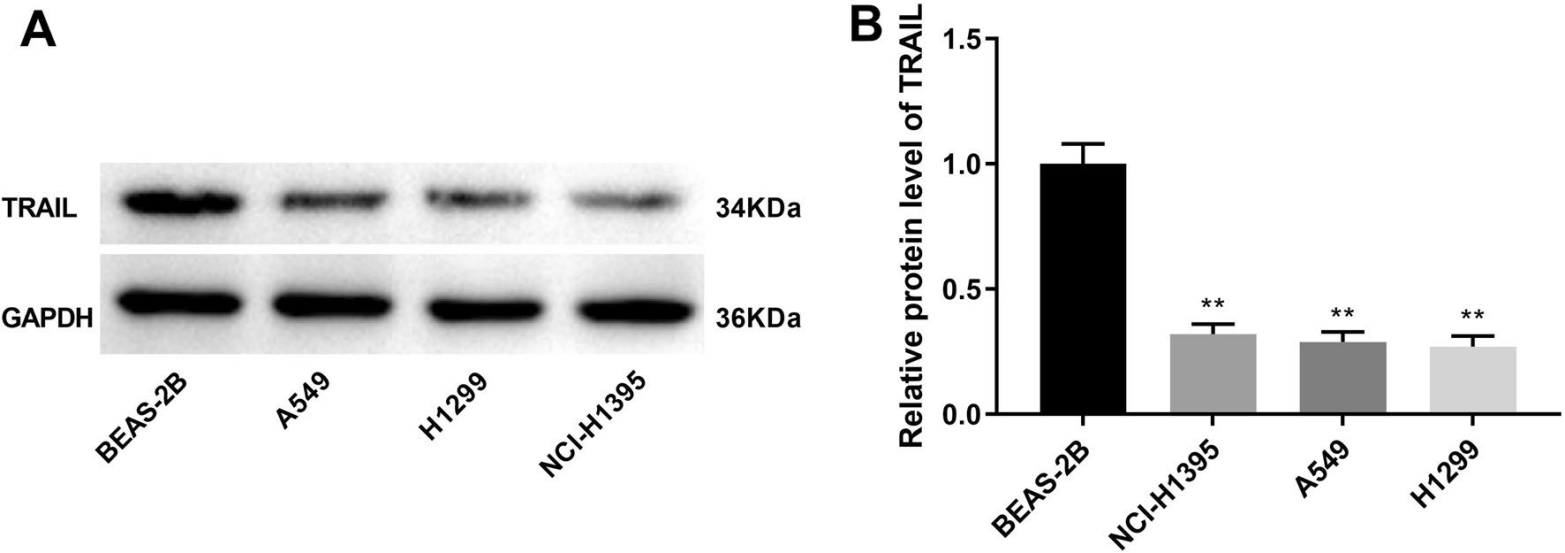
TRAIL is down-regulated in NSCLC cells. (**A**) TRAIL expression was determined in NSCLC cell lines by Western blot. (**B**) The relative protein level of TRAIL. ^**^P < 0.01 versus BEAS-2B. Data were presented as the mean ± SD, and all the experiments were performed at least three times independently

TRAIL gene expression levels were decreased in the tumor tissues of NSCLC patients [18]. We wondered whether the expression of TRAIL was downregulated in NSCLC cells. Western blot assay showed that TRAIL had a lower expression in A549, NCL-H1395, and H1299 cells compared with BEAS-2B cells (Fig. 1A-B, *P* < 0.01).

### TRAIL overexpression inhibits cell viability, migration, and invasion of NSCLC cells

**Fig. 2:**
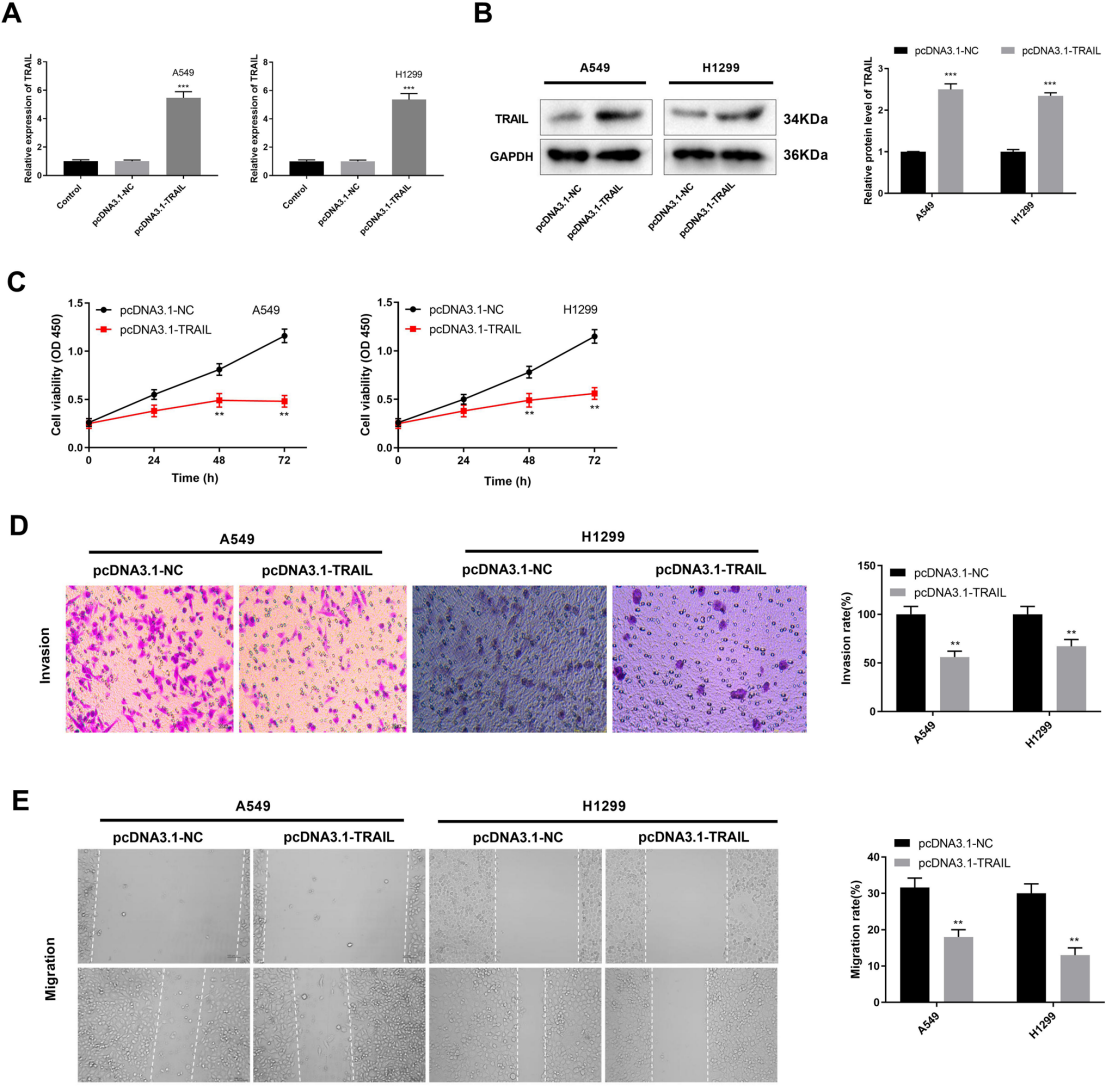
TRAIL overexpression inhibits cell viability, migration, and invasion of NSCLC cells. (**A**) TRAIL expression was detected by RT-qPCR. (**B**) TRAIL protein expression was determined by Western blot. (**C**) Cell viability was measured by CCK-8 assay. (**D**) The invasion of A549 and H1299 cells were detected by transwell assay. (**E**) The migration of A549 and H1299 cells were detected by wound healing assay. ^**^P < 0.01, ^***^P < 0.001 versus pcDNA3.1-NC. Data were presented as the mean ± SD, and all the experiments were performed at least three times independently

We investigated the function of TRAIL on NSCLC cells. TRAIL was up-regulated by transfection of pcDNA3.1-TRAIL at mRNA and protein levels in A549 and H1299 cells (Fig. 2A-B, *P* < 0.001). Compared with pcDNA3.1-NC, TRAIL overexpression reduced the viability of A549 and H1299 cells at 48 and 72 h (Fig. 2C, *P* < 0.01). Besides, overexpression of TRAIL inhibited migration and invasion of A549 and H1299 cells compared with pcDNA3.1-NC (Fig. 2D-E, *P* < 0.01).

### TRAIL overexpression predisposes ferroptosis of NSCLC cells

**Fig. 3:**
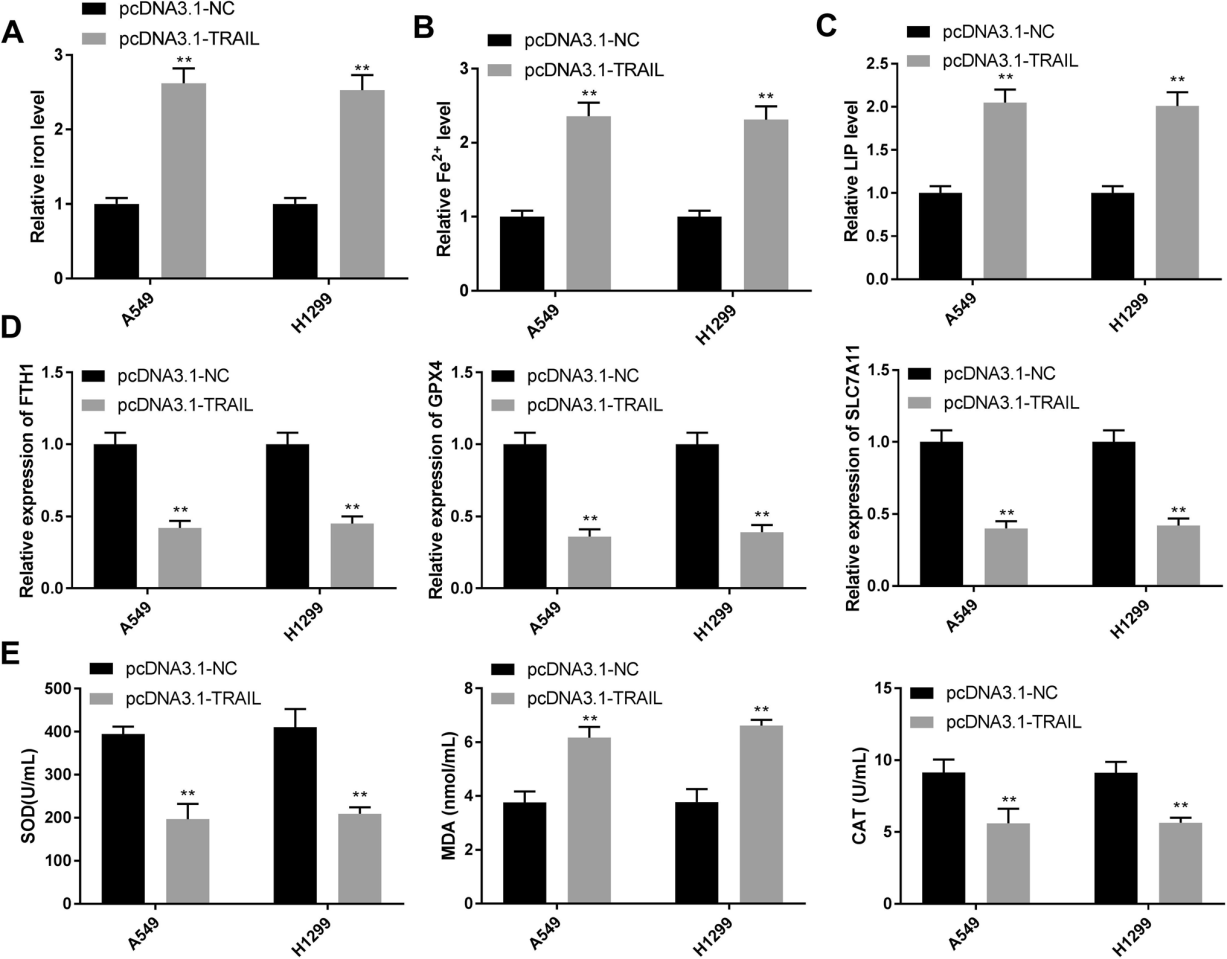
TRAIL overexpression predisposes ferroptosis of NSCLC cells.** A-B** The levels of Fe^2+^ and iron were determined by detection kits.** C** LIP level was determined by calcein-acetoxymethyl ester method.** D** The levels of ferroptosis-related genes were determined by RT-qPCR.** E** The levels of SOD, MDA, and CAT were determined by the appropriate kit. ^**^P < 0.01 versus pcDNA3.1-NC. Data were presented as the mean ± SD, and all the experiments were performed at least three times independently

Studies have reported that ferroptosis plays a key role in the development of cancer. Iron metabolism and lipid peroxidation are considered important causes of ferroptosis. As shown in Fig. 3A-C, the levels of iron, Fe^2+^, and LIP were increased by TRAIL overexpression in A549 and H1299 cells compared with pcDNA3.1-NC (*P* < 0.01). Ferroptosis-associated proteins FTH1, GPX4, and SLC7A11 are involved in the regulation of the progression of ferroptosis [19–21]. Next, we detected the expression of FTH1, GPX4, and SLC7A11 in A549 and H1299 cells. Compared with the pcDNA3.1-NC group, the levels of FTH1, GPX4, and SLC7A11 were reduced in the pcDNA3.1-TRAIL group (Fig. 3D, *P* < 0.01). In addition, TRAIL overexpression enhanced MDA level, while reduced the levels of SOD and CAT in A549 and H1299 cells compared with the pcDNA3.1-NC group (Fig. 3E, *P* < 0.01). The results indicated that TRAIL overexpression might induce ferroptosis of NSCLC cells.

### TRAIL interacts with the ASK-1/JNK1 pathway

**Fig. 4:**
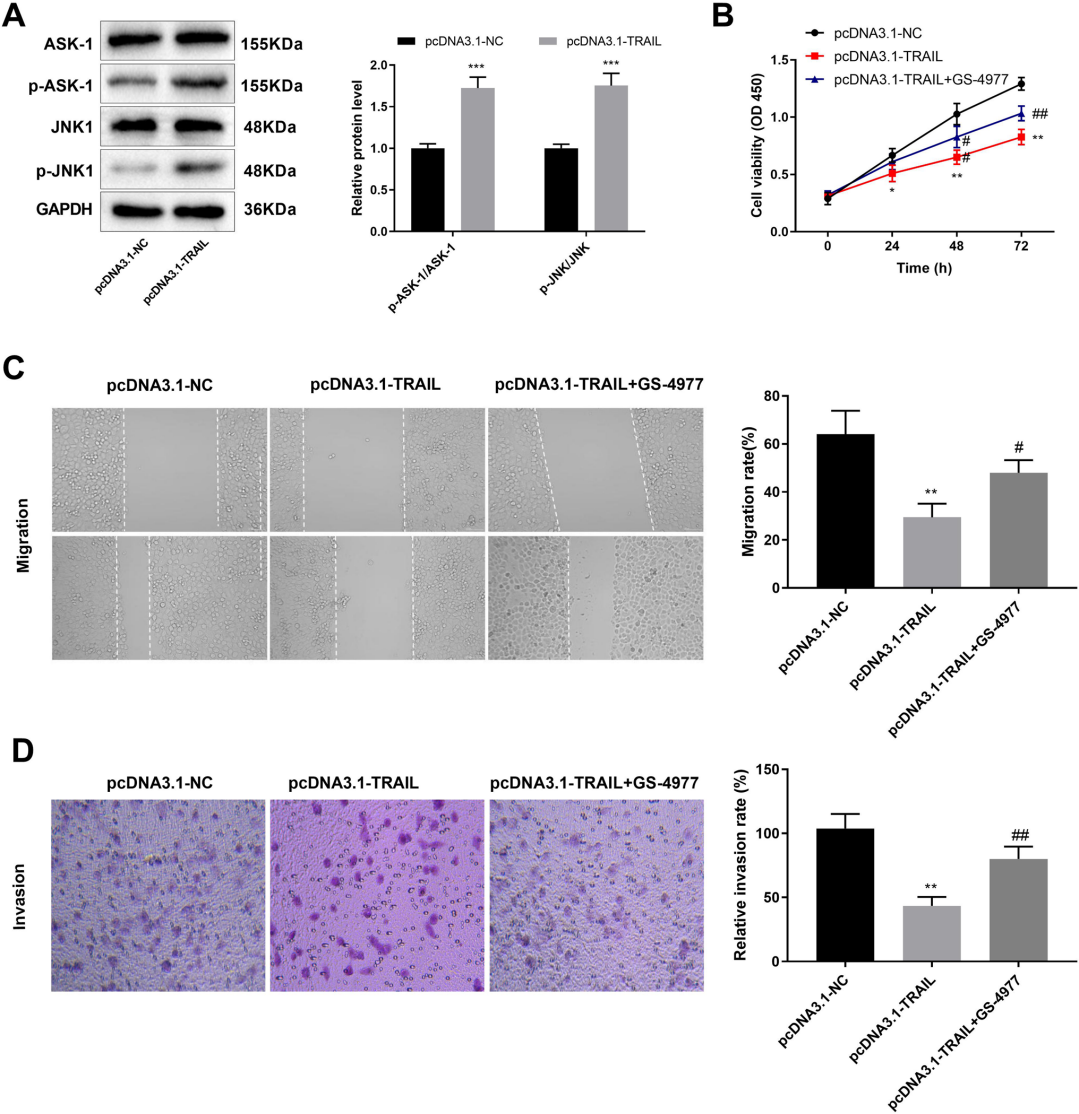
TRAIL interacts with the ASK-1/JNK1 pathway. (**A**) The expression of ASK-1, JNK1, p-ASK-1, and p-JNK1 was detected by western blot. (**B**) Cell viability was detected by MTT assay. (**C**) Wound healing was used to determine cell migration of H1299 cells. (**D**) The invasion of H1299 cells were detected by transwell assay. ^**^P < 0.01, ^***^P < 0.001 versus pcDNA3.1-NC. ^#^*P* < 0.05, ^##^*P* < 0.01 versus pcDNA3.1-TRAIL. Data were presented as the mean ± SD, and all the experiments were performed at least three times independently

The ASK-1/JNK pathway plays an important role in the development of tumors, such as lung cancer [22, 23]. Western blot assay showed that ASK1/JNK1 pathway was activated, evidenced by increase of the phosphate modification of ASK-1 and JNK after transfection of pcDNA3.1-TRAIL (Fig. 4A, *P* < 0.01). To explore the regulatory mechanism of TRAIL on NSCLC development, H1299 cells were treated with ASKI-1 inhibitor (GS-4977, 10 µM). In the pcDNA3.1-TRAIL + GS-4977 group, the viability, migration, and invasion of H1299 cells were increased compared with the pcDNA3.1-TRAIL group (Fig. 4B-D, *P* < 0.05). The results suggested that GS-4977 attenuated the inhibition effect of TRAIL overexpression on H1299 cells.

### GS-4977 attenuates the effects of TRAIL overexpression on ferroptosis in H1299 cells

**Fig. 5:**
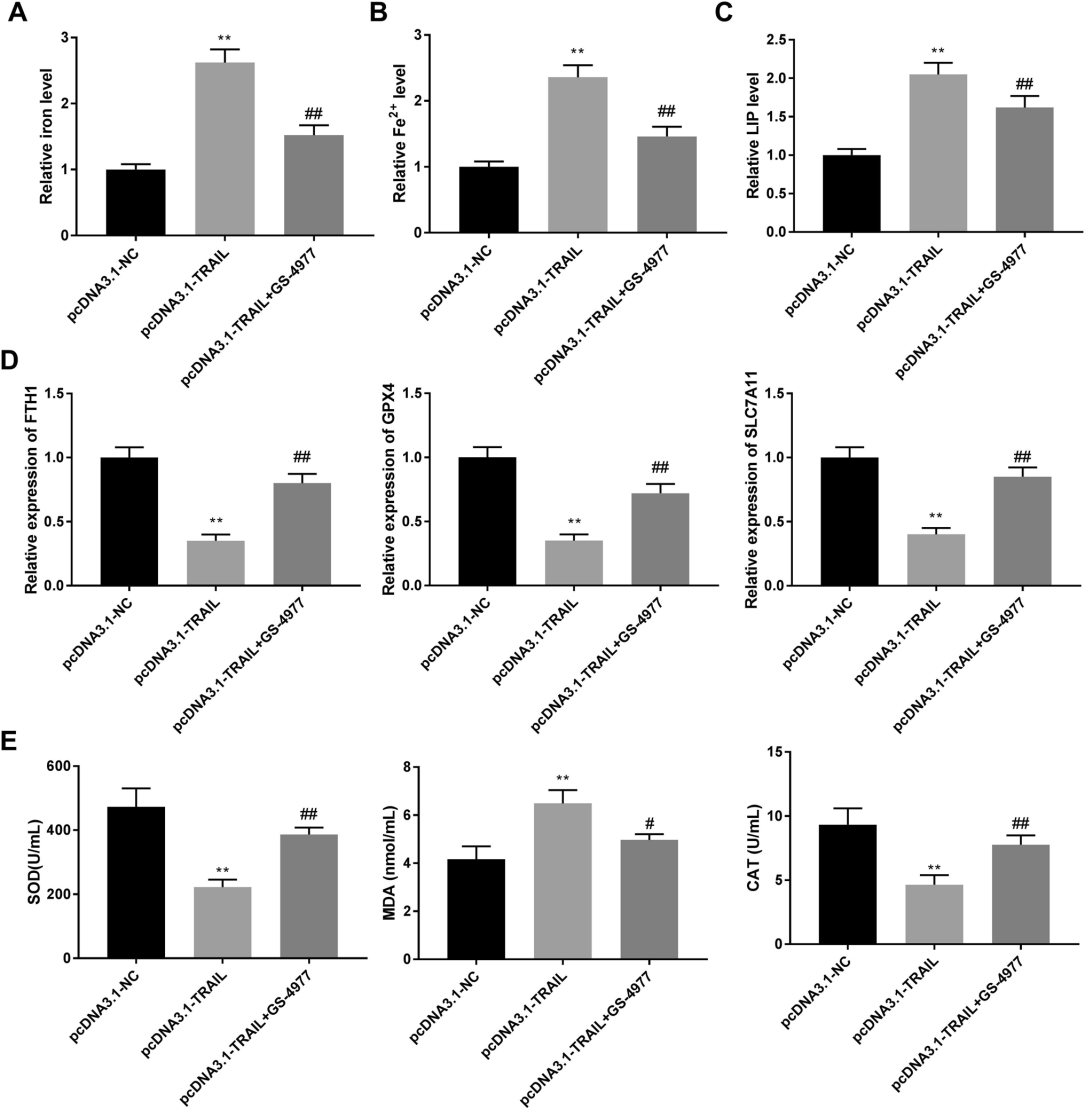
GS-4977 attenuates the effects of TRAIL overexpression on ferroptosis in H1299 cells. A-B. The levels of Fe^2+^ and iron were determined by detection kits. C. LIP level was determined by calcein-acetoxymethyl ester method. D. The expression of ferroptosis-related genes was determined by RT-qPCR. E. The levels of SOD, MDA, and CAT were determined by the appropriate kit. ^**^P < 0.01 versus pcDNA3.1-NC. ^#^*P* < 0.05, ^##^*P* < 0.01 versus pcDNA3.1-TRAIL. Data were presented as the mean ± SD, and all the experiments were performed at least three times independently

To investigate whether ASK-1/JNK1 pathway was involved in TRAIL-induced ferroptosis, ferroptosis-related marks were detected after H1299 cells were treated with ASKI-1 inhibitor GS-4977. The levels of total iron, Fe^2+^, and LIP were decreased in the pcDNA3.1-TRAIL + GS-4977 group compared with the pcDNA3.1-TRAIL group (Fig. 5A-C, *P* < 0.01). GS-4977 reduced the inhibition effects of TRAIL overexpression on FTH1, GPX4, and SLC7A11 in H1299 cells compared with the pcDNA3.1-TRAIL group (Fig. 5D, *P* < 0.01). Furthermore, as shown in Fig. 5E, MDA level was reduced (*P* < 0.05), while the levels of SOD and CAT were increased in the pcDNA3.1-TRAIL + GS-4977 group compared with the pcDNA3.1-TRAIL group (*P* < 0.01). The data suggested that GS-4977 attenuated the effects of TRAIL overexpression on ferroptosis of NSCLC cells.

### TRAIL overexpression inhibits tumor growth and promotes ferroptosis in vivo

**Fig. 6:**
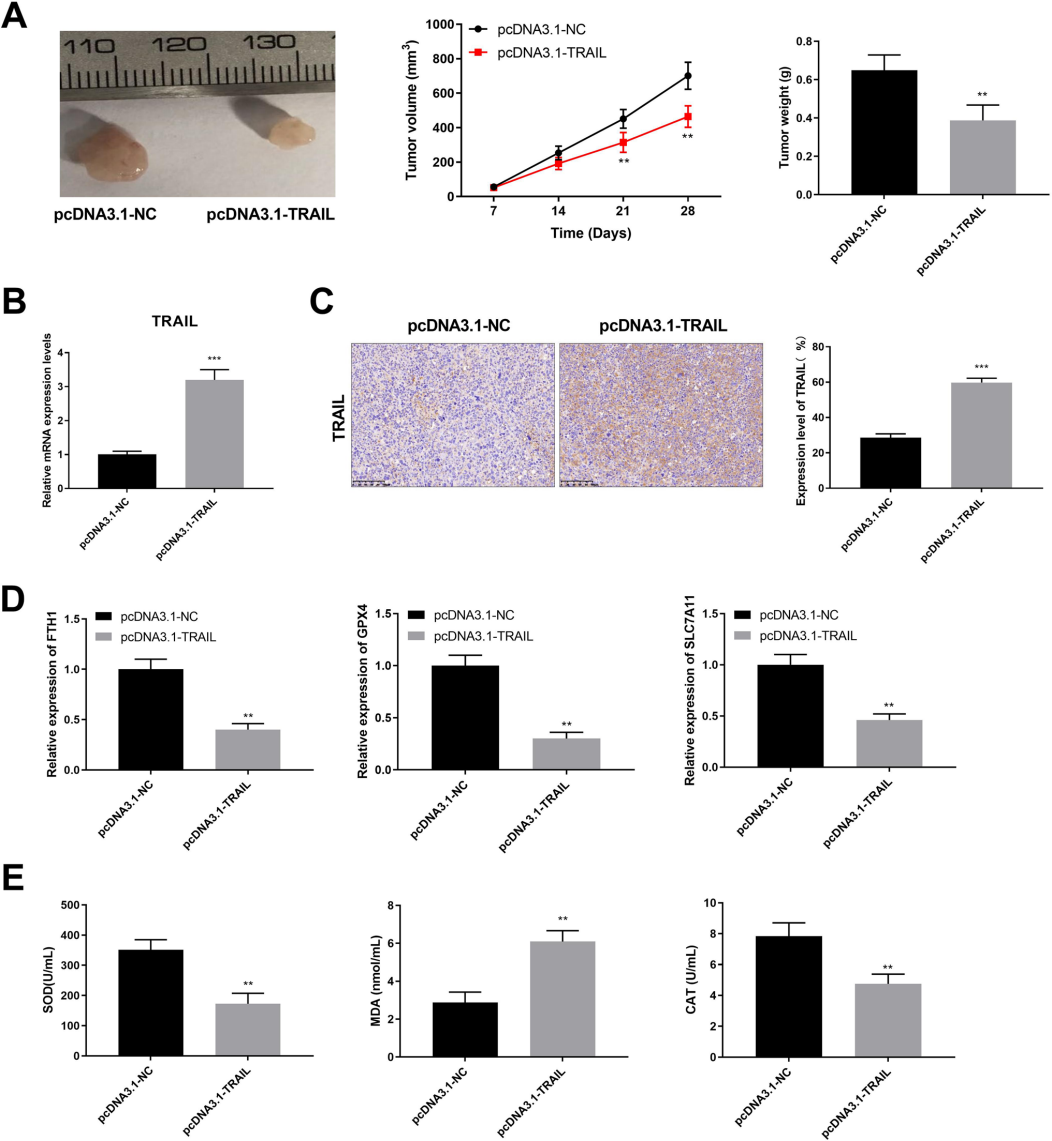
TRAIL overexpression inhibits tumor growth and promotes ferroptosis in vivo. (**A**) Tumor volume and weight were measured in different groups (*n* = 6 for each condition). (**B**) The relative mRNA expression level of TRAIL was detected through RT-qPCR. (**C**) IHC analysis of TRAIL for tissues of xenograft tumors. Scale bar: 100 μm. (**D**) The expression of ferroptosis-related genes was determined by RT-qPCR. (**E**) The levels of SOD, MDA, and CAT were detected by the appropriate kit. ^**^P < 0.01, ^***^P < 0.001 versus pcDNA3.1-NC. Data were presented as the mean ± SD.

The role of TRAIL was further explored in the tumor xenograft model. TRAIL overexpression suppressed tumor volume and weight compared with the pcDNA3.1-NC group (Fig. 6A, *P* < 0.01). We found the relative mRNA expression level of TRAIL was elevated in the pcDNA3.1- TRAIL group (Fig. 6B, *P* < 0.001). The protein expression levels of TRAIL in the TRAIL overexpression group was significantly higher than that in the pcDNA3.1-NC group, as confirmed using IHC (Fig. 6C, *P* < 0.001). Furthermore, the effect of TRAIL on ferroptosis is further demonstrated in vivo. Compared with the pcDNA3.1-NC group, increased TRAIL reduced the expression of FTH1, GPX4, and SLC7A11 in tumor tissues (Fig. 6D, *P* < 0.01). TRAIL overexpression increased MDA, while reduced the levels of SOD and CAT in tumor xenograft model compared with the pcDNA3.1-NC group (Fig. 6E, *P* < 0.01). The results showed that TRAIL overexpression contributed to inhibiting tumor growth and promoted ferroptosis.

## Discussion

NSCLC is one of the leading causes of cancer death. Despite advances in the treatment of NSCLC, patient prognosis is poor [24]. Therefore, it is necessary to develop new therapeutic targets to improve clinical outcomes in NSCLC patients through continuous research. In this study, we reported that TRAIL was down-regulated in NSCLC cells, and exerted anti-tumor effects in vivo and in vitro. Mechanistically, TRAIL could induce ferroptosis through the activation of ASK-1/JNK1 pathway.

TRAIL has attracted great attention due to its ability to induce apoptosis in tumor cells [25]. Studies have reported that TRAIL is down-regulated in a variety of cancers, such as myeloid leukemia, colorectal cancer, and pancreatic cancer [26–28]. Congruously, in this study, TRAIL has a lower expression level in NSCLC cells. TRAIL gene delivery has been shown to prevent the growth of tumor cells, such as liver cancer, pancreatic cancer, colon cancer, and glioblastoma [29]. The up-regulation of TRAIL expression inhibits the G2/M cell cycle of colon tumor cells and suppresses the tumor growth in xenograft mice models [30]. TRAIL induces apoptosis in broad-spectrum tumor cell lines without affecting normal cells, implying that TRAIL may be a promising anti-tumor agent [31]. TRAIL induces apoptosis and reduces cell viability, thereby inhibiting lung cancer cell growth [32]. TRAIL can also suppress metastasis and invasion of colon cancer cells by reducing the release of TGF-β1 and promoting platelet apoptosis [33]. Therefore, we speculated TRAIL may inhibit the growth of NSCLC cells. Consistent with previous studies, the up-regulation of TRAIL inhibited NSCLC cell viability, migration, and invasion. Meanwhile, TRAIL suppressed tumor growth in NSCLC xenograft models. Consequently, our findings indicated that TRAIL may exert an anti-tumor effect in NSCLC.

Ferroptosis is a form of cell death caused mainly by lipid peroxidation and excessive accumulation of intracellular iron [34]. In this study, TRAIL up-regulation increased the levels of iron, LIP, Fe^2+^, and MDA, as well as reduced the levels of ferroptosis-related genes FTH1, GPX4, and SLC7A11 of NSCLC cells. Our results indicated that TRAIL overexpression promoted the ferroptosis of NSCLC cells. The promotion of ferroptosis contributes to inhibiting tumor development, and targeting ferroptosis may be an effective and selective approach to cancer therapy [35–37]. The overexpression of SLC7A11 promotes tumor growth partly by inhibiting ferroptosis [20]. Baicalin exerts an anti-bladder cancer function by inhibiting FTH1 to induce ferroptosis [38]. The silencing of GPX4 can enhance the anti-cancer effect of Lapatinib via promoting ferroptosis of NSCLC cells, thereby inhibiting the development of NSCLC [39]. Therefore, we speculated that TRAIL may inhibit NSCLC development by regulating ferroptosis. Furthermore, the combination of ferroptosis inducer with TRAIL can strongly enhance its inhibitory effect on tumor development [40]. Taken together, the up-regulation of TRAIL may be a potential therapeutic strategy for NSCLC by promoting ferroptosis.

c-Jun N-terminal kinase (JNK) is a member of the mitogen-activated protein kinase (MAPK) family, and plays a key role in various types of cancers, including gastric, liver, and breast cancers [41]. ASK1/JNK signaling pathway is involved in the development of multiple diseases, including cancer [42–44]. In this study, we found that TRAIL activated the ASK-1/JNK1 pathway in NSCLC cells. Previous studies have suggested ASK-1/JNK1 pathway is involved in the regulation of tumor development. For instance, the up-regulation of JNK1 in Treg cells contributes to inhibiting the tumor growth of breast cancer model mice [45]. Ginsenoside induces ROS accumulation and activates the ASK-1/JNK pathway, thereby inducing apoptosis of gastric cancer cells [42]. Chaetocin regulates ROS-mediated ASK-1/JNK pathways to induce apoptosis of intrahepatic cholangiocarcinoma cells and arrest the cell cycle [43]. Down-regulation of Trx1 activated ASK and triggered ASK-JNK or ASK-p38 pathway to induce apoptotic cell death in lung cancer [46]. Therefore, we speculated that TRAIL may interact with the ASK-1/JNK1 pathway to regulate NSCLC development. In the present study, ASKI-1 inhibitor GS-4977 attenuated the inhibitory effects of TRAIL up-regulation on the viability, invasion, migration, and ferroptosis of NSCLC cells. Taken together, we indicated that TRAIL may mediate ferroptosis in NSCLC through regulating the ASK-1/JNK1 pathway.

## Conclusion

In summary, in this research, we investigated the impact of TRAIL on A549 and H1299 cells. These results suggested that TRAIL could inhibit cell viability, invasion, and migration of NSCLC cells. In vivo experiments further demonstrated that TRAIL inhibited tumor growth in mice. In addition, TRAIL predisposed NSCLC cells to ferroptosis in vitro and in vivo by regulating the ASK-1/JNK1 pathway. Our findings may provide novel light on possible therapeutic strategies for the treatment of NSCLC.

### Electronic supplementary material

Below is the link to the electronic supplementary material.

Supplementary Material 1

## Data Availability

All data in the manuscript is available through the responsible corresponding author.
