# Supplementary material for: TRAIL predisposes non-small cell lung cancer to ferroptosis by regulating ASK-1/JNK1 pathway
Source: Discov Oncol. 2024 Feb 21;15:45. doi: 10.1007/s12672-024-00890-9 (PMC10881944; doi:10.1007/s12672-024-00890-9)

Figure 1 GAPDH

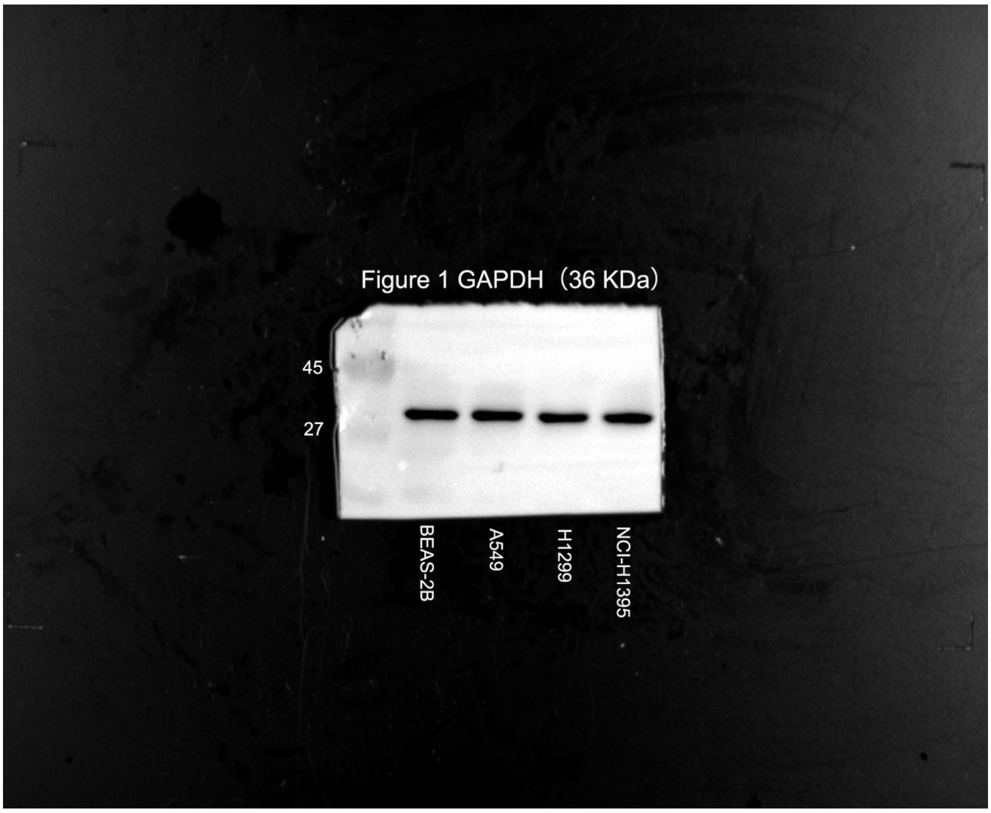

Figure 1 TRAIL

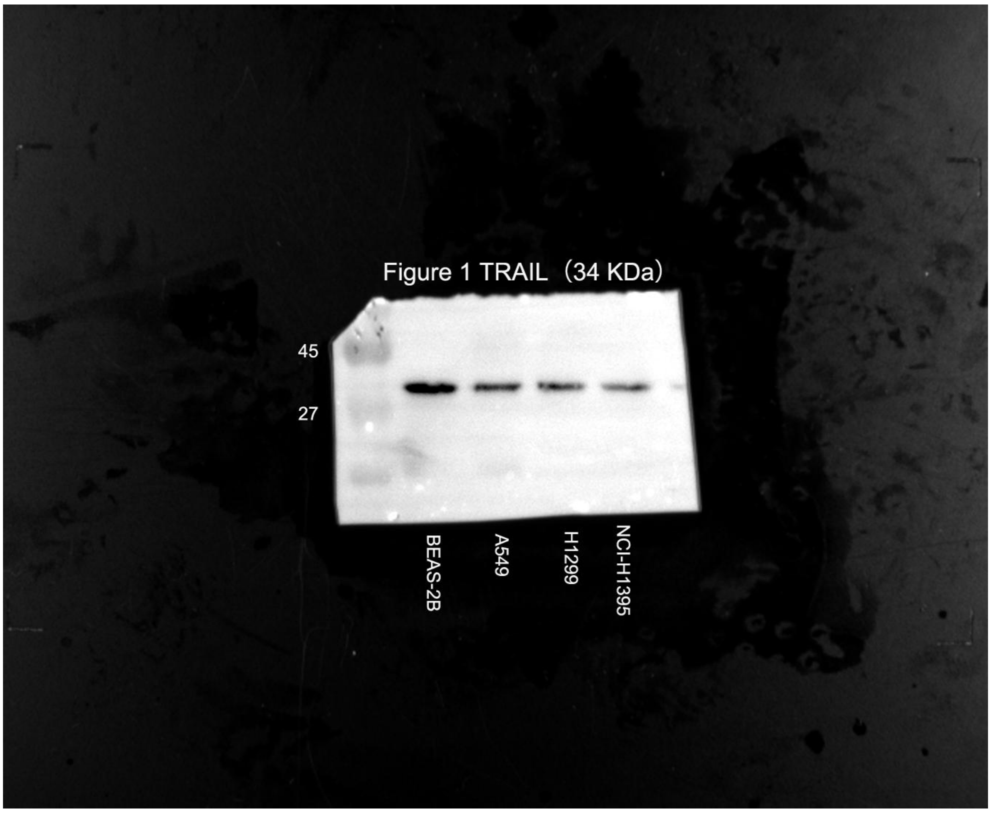

Figure 2 GAPDH（A549）

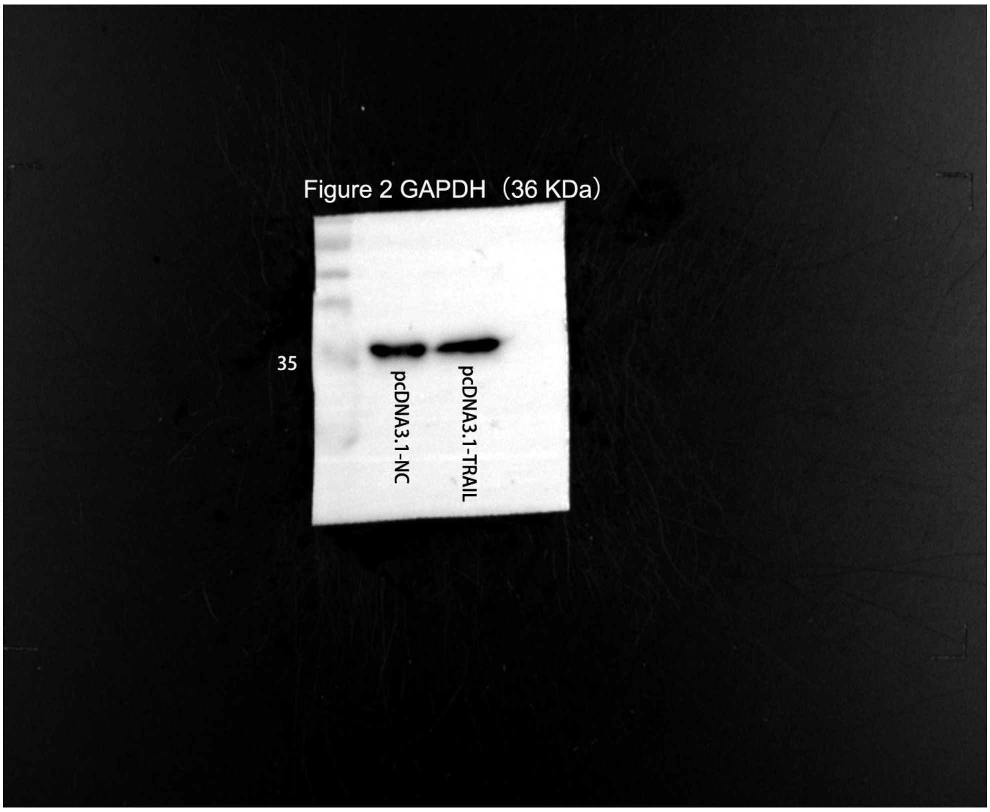

Figure 2 TRAIL（A549）

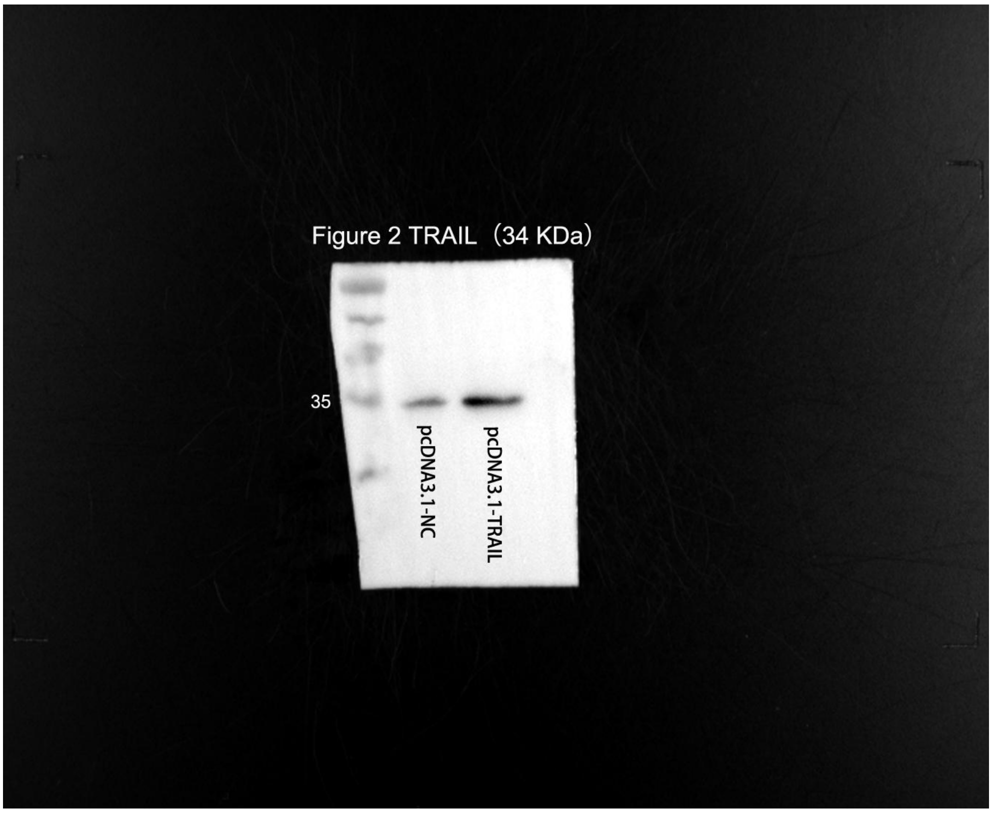

Figure 2 GAPDH（H1299）

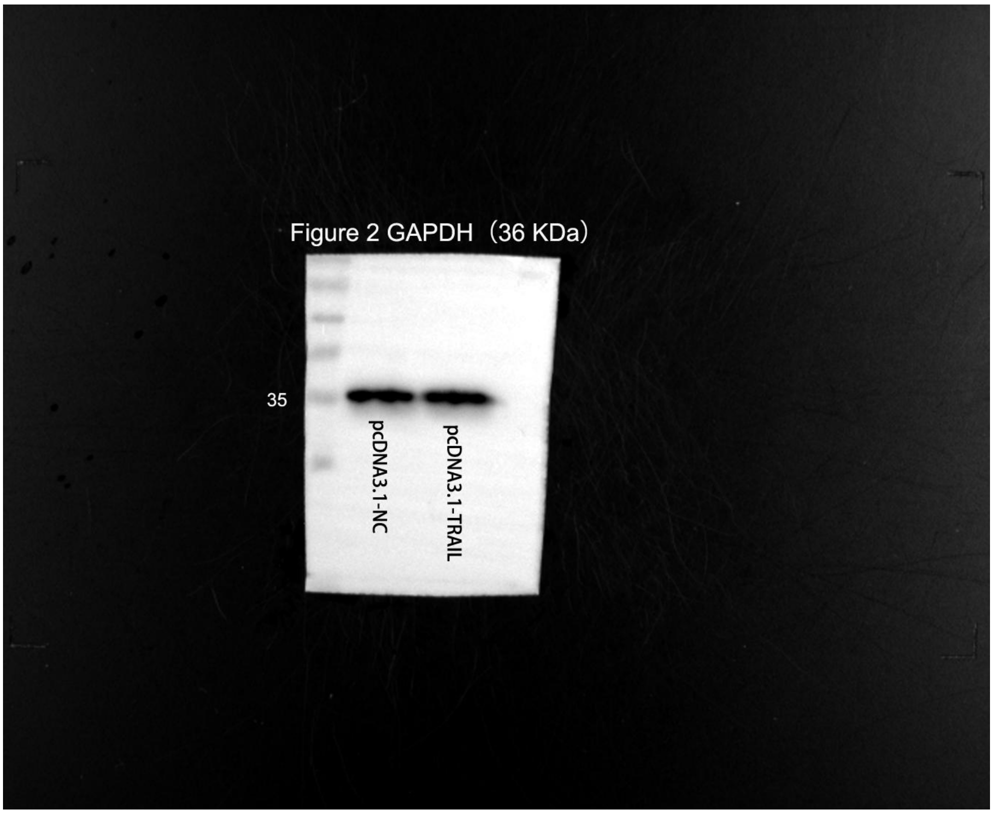

Figure 2 TRAIL（H1299）

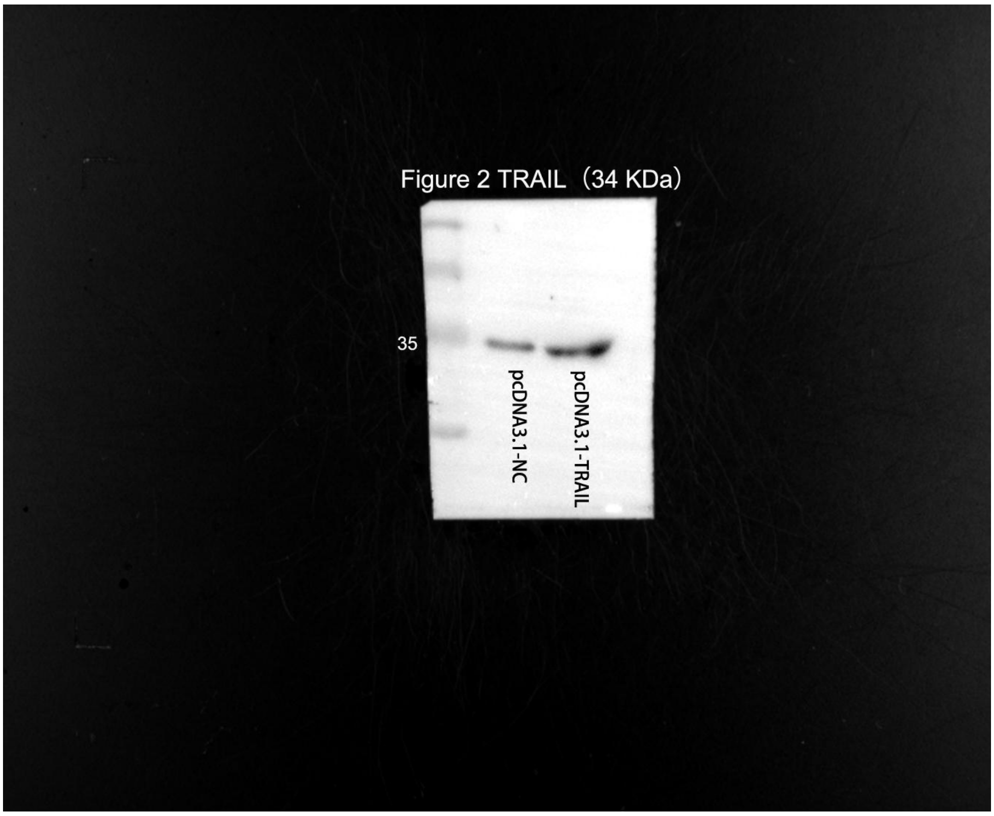

Figure 4 ASK-1

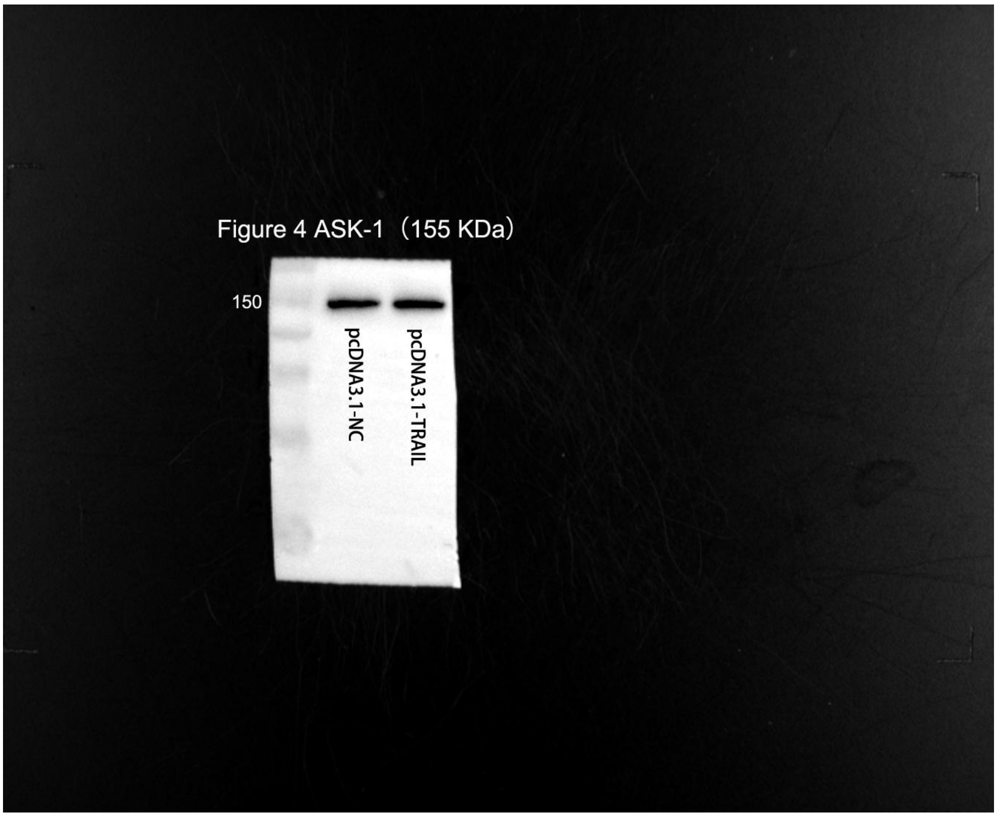

Figure 4 p-ASK-1

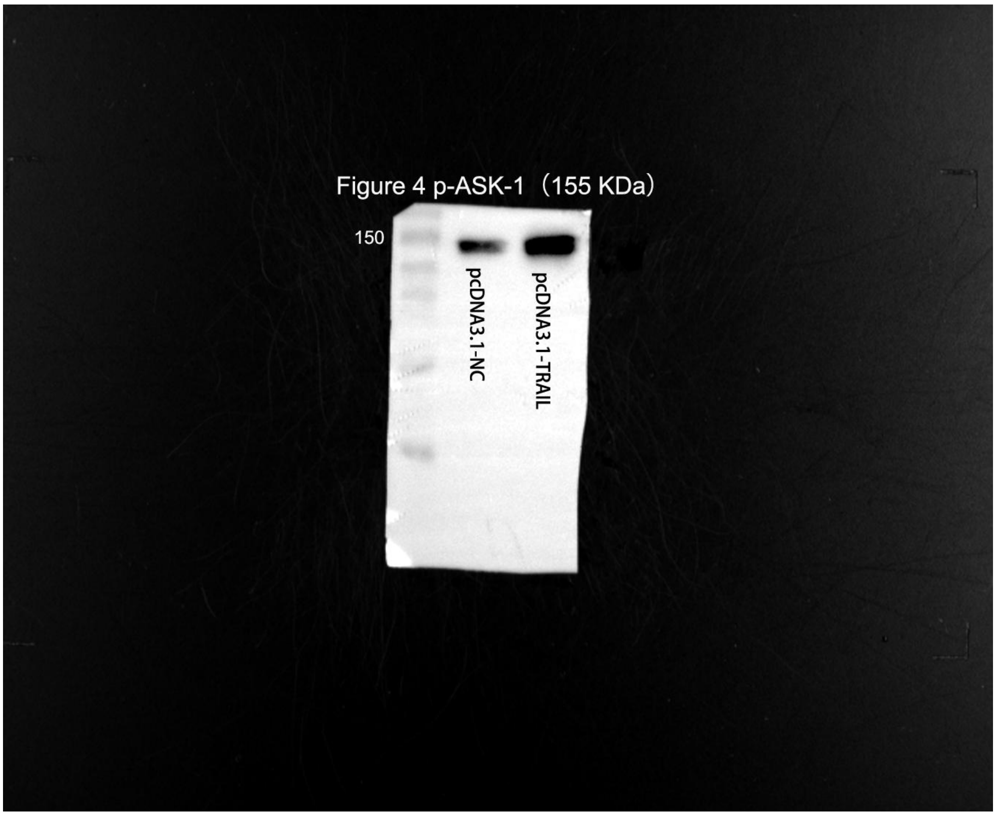

Figure 4 JNK-1

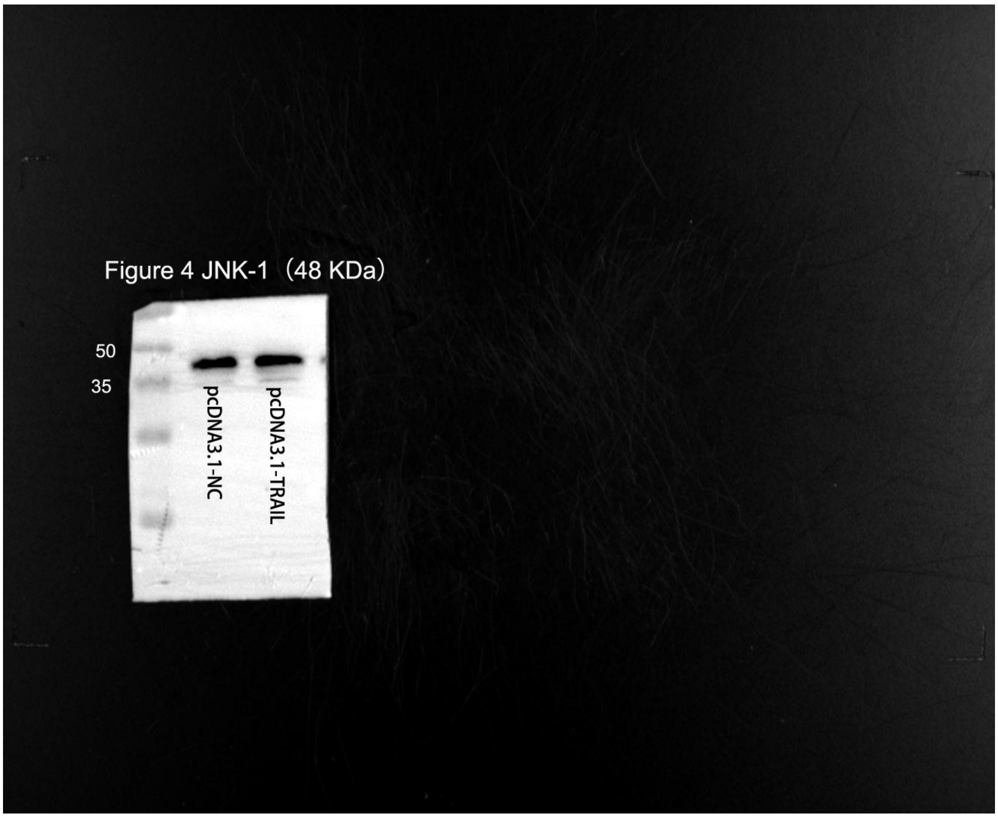

Figure 4 p-JNK-1

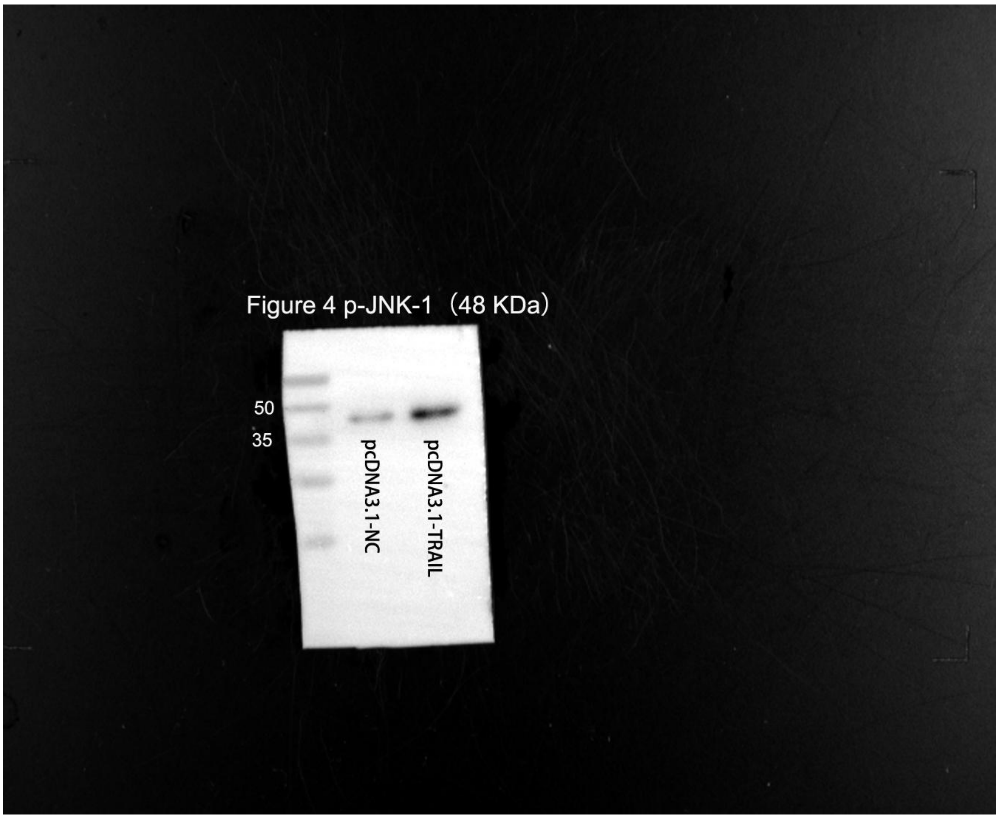

Figure 4 GAPDH

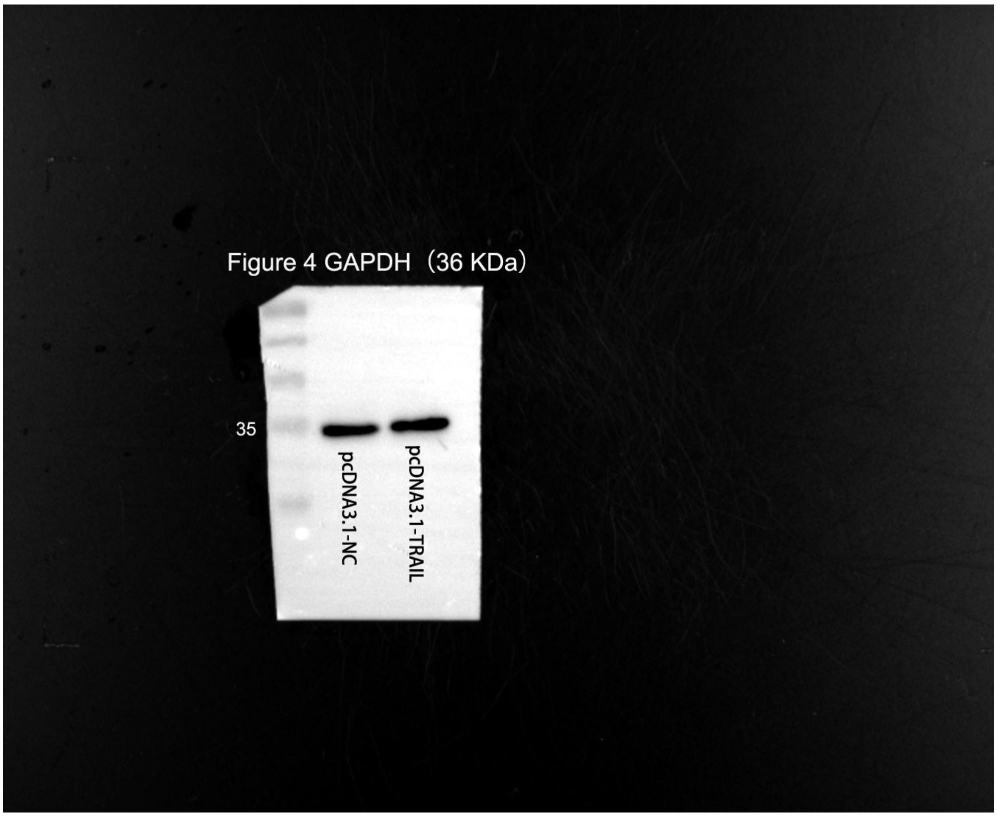

Supplement: Supplementary file 1 — Supplementary Material 1 [file 12672_2024_890_MOESM1_ESM.pdf]
